# A Dual-Recognition Electrochemical Sensor Using Bacteria-Imprinted Polymer and Concanavalin A for Sensitive and Selective Detection of *Escherichia coli* O157:H7

**DOI:** 10.3390/foods14071099

**Published:** 2025-03-21

**Authors:** Xuejie Niu, Yuanbing Ma, Hui Li, Shuang Sun, Luoyuan Shi, Juan Yan, Donglei Luan, Yong Zhao, Xiaojun Bian

**Affiliations:** 1 International Research Center for Food and Health, College of Food Science and Technology, Shanghai Ocean University, Shanghai 201306, China; 15660694845@163.com (X.N.); y15735352558@163.com (Y.M.); huili_shou@163.com (H.L.); shuangsun01@163.com (S.S.); 15737912061@163.com (L.S.); j-yan@shou.edu.cn (J.Y.); dlluan@shou.edu.cn (D.L.); yzhao@shou.edu.cn (Y.Z.); 2 Laboratory of Quality and Safety Risk Assessment for Aquatic Product on Storage and Preservation (Shanghai), Ministry of Agriculture and Rural Affairs, Shanghai 201306, China; 3 State Key Laboratory of Transducer Technology, Shanghai Institute of Microsystem and Information Technology, Chinese Academy of Sciences, Shanghai 200050, China

**Keywords:** electrochemical sensors, pathogenic bacteria, bacteria-imprinted polymer, concanavalin A

## Abstract

The accurate detection and quantification of pathogenic bacteria is crucial for ensuring public health. In this work, we propose a sensitive and selective sandwich electrochemical sensor for detecting *Escherichia coli* O157:H7 (*E. coli* O157:H7). The sensor employs a dual-recognition strategy that combines a bacteria-imprinted polymer (BIP) and concanavalin A (ConA). The BIP is formed in situ on the electrode surface as the capture probe, while gold nanoparticles co-functionalized with ConA and the electroactive molecule 6-(ferrocenyl)hexanethiol (Au@Fc-ConA) serve as the signaling probe. When *E. coli* O157:H7 is present, the bacteria are selectively captured by the BIP. The captured bacteria interact with Au@Fc-ConA through ConA’s sugar-binding properties, triggering Fc oxidation and generating a current proportional to the bacterial concentration. The sensor exhibits a linear detection range of 10^1^–10^5^ CFU mL^−1^ and a low detection limit of 10 CFU mL^−1^. Additionally, it demonstrates high sensitivity in complex milk samples, detecting *E. coli* O157:H7 at concentrations as low as 10 CFU mL^−1^, with recoveries ranging from 94.16% to 110.6%. Even in the presence of a 100-fold higher concentration of *E. coli* O6, the sensor effectively distinguishes *E. coli* O157:H7 from it. Moreover, it exhibits high reproducibility with a relative standard deviation of 2%. This study proposes a unique dual recognition strategy that combines simplicity and high performance. This method enables the selective detection of *E. coli* O157:H7 in real samples, providing a promising tool for food safety monitoring.

## 1. Introduction

Recently, food safety has become a global priority, playing a crucial role in nutrition, sustainable food production, and social stability [1,2]. However, contamination by foodborne pathogenic bacteria poses a growing threat to both food security and public health [3,4,5,6]. Among these pathogens, *Escherichia coli* O157:H7 (*E. coli* O157:H7) has emerged as a particularly significant threat [7]. It is a major cause of foodborne diarrheal diseases and can lead to severe complications, including respiratory infections, urinary tract infections, pneumonia, and other life-threatening conditions [8]. Each year, *E. coli* O157:H7 is responsible for approximately 265,000 infections, resulting in substantial societal and economic burdens [9]. Therefore, there is an urgent need for a rapid, reliable, and straightforward method for detecting pathogenic bacteria to safeguard public health and ensure food safety.

Pathogenic bacteria are a major cause of foodborne illnesses, underscoring the necessity for routine screening to mitigate risks and ensure food safety. The standard plate counting method [10] is widely used but is time-consuming [11]. Alternative bacterial detection techniques, such as enzyme-linked immunosorbent assay (ELISA) [12], mass spectrometry [13], and polymerase chain reaction (PCR) [14], offer high sensitivity and accuracy. However, these methods require specialized equipment, trained personnel, and extensive sample preparation. Moreover, they are prone to false positives and false negatives, compromising detection reliability [15]. Consequently, there is an urgent need for rapid, sensitive, and portable detection techniques to enable efficient and reliable identification of harmful bacteria.

Compared to traditional methods, electrochemical sensors have gained increasing popularity for detecting pathogenic bacteria due to their rapid response time, affordability, ease of use, and potential for miniaturization [16]. These sensors convert chemical information, such as proton release or electron transfer, into measurable signals like current or potential fluctuations, enabling efficient analysis [17]. The recognition element (receptor) plays a crucial role in determining the sensor’s selectivity and sensitivity [18]. Common recognition elements include antibodies [19], aptamers [20], antimicrobial peptides [21], molecularly imprinted polymers [22], and boronic acid affinity compounds [23]. While antibodies offer high specificity and affinity [24], they face challenges such as high costs, limited stability, and ethical concerns related to animal testing [25]. Therefore, developing synthetic receptors as alternatives to antibodies is essential.

Molecularly imprinted polymers (MIPs), often termed “synthetic antibodies”, serve as promising alternatives to natural antibodies [26]. They possess artificial binding sites that selectively recognize target molecules [27], offering benefits such as cost-effectiveness, straightforward preparation, and high physical and chemical stability. By enhancing the specificity of electrochemical sensors, MIPs effectively bridge the gap between natural and synthetic antibodies [28]. With these unique properties, MIPs are increasingly used in electrochemical techniques for whole bacterial cell detection. Concanavalin A (ConA) is a plant lectin derived from *Canavalia ensiformis* that specifically binds to sugar molecules such as α-D-mannose, α-D-glucose, and their related structures [29]. The surface of *E. coli* O157:H7 is rich in polysaccharides, including lipopolysaccharides (LPS), whose O-antigen chains may contain mannose or glucose residues recognized by ConA [30,31,32].

Upon interaction, ConA specifically binds to these sugar molecules, forming stable ligand complexes due to its multiple binding sites. This enables ConA to simultaneously bind multiple bacterial cells, inducing cross-linking and bacterial agglutination, making it suitable for detecting *E. coli* O157:H7 [33,34]. Studies have shown that ConA-mediated agglutination is a reliable method for detecting *E. coli* O157:H7 [35], highlighting ConA’s potential in developing effective detection methods for pathogenic bacteria. Meanwhile, the rapid advancement of nanomaterials has led to their widespread application [36], and integrating them with sensors can greatly enhance both the sensitivity and selectivity [37,38,39].

However, electrochemical sensors based on bacterial imprinting polymers (BIP), such as the one developed by a previous team [40], offer high selectivity and sensitivity for detecting *E. coli* O157:H7, with a limit of quantification as low as 10^3^ CFU mL^−1^. However, these sensors struggle to detect low bacterial concentrations (<10^3^ CFU mL^−1^) in real samples. In contrast, an aptamer-based sensor [41] improves detection performance due to the high specificity of aptamers, but its complex and costly design limits widespread application. However, the electrochemical sensor based on ConA address these issues by enhancing low-concentration detection, improving specificity, and providing a reliable, rapid solution compared to BIP- and aptamer-based methods.

In this study, we developed a highly specific and sensitive sandwich-type electrochemical sensor for detecting *E. coli* O157:H7, which is achieved through the dual recognition strategy of bacterial imprinting polymer (BIP) and ConA. The BIP is directly immobilized on the electrode surface through electro-polymerization as the capture probe. ConA and 6-(ferrocenyl)hexanethiol (Fc) are conjugated to gold nanoparticles (Au NPs) through Au-S bonds. This forms a signaling nanoprobe (Au@Fc-ConA). When target bacteria are present, they are selectively captured by the BIP. The signaling nanoprobe (Au@Fc-ConA) then binds to the captured bacteria, triggering an electrochemical response through the oxidation of Fc [18]. The current increases with bacterial concentration, enabling the quantification of *E. coli* O157:H7. The sensor’s performance, including its linear range, detection limit, selectivity, reproducibility, and stability, was thoroughly evaluated. Its practical applicability was demonstrated by detecting target bacteria in milk following a simple 10-fold dilution pre-treatment. Compared to traditional methods like PCR [42], our electrochemical sensor offers significant advantages, including simpler preparation, reduced time, and minimal pre-treatment in real-world samples like milk. The dual recognition mechanism of BIP and ConA enhances specificity and sensitivity, making it an ideal tool for the rapid detection of foodborne pathogens in complex matrices.

## 2. Materials and Methods

### 2.1. Reagents

The bacterial species employed in this study were *E. coli* O157:H7 (ATCC 43889), *Salmonella paratyphi B* (*S. Paratyphi B*, CMCC 50094), *Staphylococcus aureus* (*S. aureus*, ATCC 25923), *Listeria monocytogenes* (*L. monocytogenes*, ATCC 19115) and *Escherichia coli* O6 (*E. coli* O6, ATCC 25922). Concanavalin A (ConA) (25 mg, water: 1% (*w*/*v*)) and 6-(ferrocenyl)hexanethiol (Fc) (molecular weight: 302.26, 250 mg) were procured from Sigma-Aldrich (Shanghai, China). Additional reagents included 3-thiopheneethanol (TE) (97%+, molecular weight: 128.19), hydroxychloroauric acid (HAuCl₄·xH₂O) (99%, Au: 50%), trisodium citrate (AR grade, molecular weight: 294.10, ≥99.0%), acetic acid (HAc) (AR grade, ≥99.5%), formaldehyde (BC grade, 30–40%), and thioglycolic acid (TGA) (molecular weight: 92.12, 95%) from Adamas Reagents Co., Ltd. (Shanghai, China); ethanol (AR grade, ≥99.7%), n-hexane (AR grade, ≥97.0%), KCl (AR grade, ≥99.5%), K_4_[Fe(CN)_6_] (molecular weight: 422.39, 98%+), and K_3_[Fe(CN)₆] (AR grade, ≥99.5%) from Shanghai Titan Technology Co. (Shanghai, China); hexyltrimethylammonium bromide (CTAB) (molecular weight: 364.45, 99%) from BBI Life Sciences (Shanghai, China); Tris-HCl (1 mol/L, pH 8.0) buffer solution from Shanghai Yuanye Biotechnology Co., Ltd. (Shanghai, China); and 4-(4,6-Dimethoxy-1,3,5-triazin-2-yl)-4-methylmorpholinium chloride (DMTMM) (25 g, molecular weight: 276.72) from Shanghai Haoyuan Biomedical Technology Co., Ltd. (Shanghai, China).

### 2.2. Instruments and Test

Electrochemical tests were made through the CHI 660E workstation, which relied on three electrodes. (Working electrode: glassy carbon electrode (GCE, 3 mm in diameter); reference electrode: saturated calomel electrode (SCE); counter electrode: platinum sheet). To assess interfacial charge transfer, electrochemical impedance spectroscopy (EIS) measurements were performed in 0.1 M KCl with [Fe (CN)_6_]^3−/4−^ (1 mM) under open-circuit conditions (amplitude: 5 mV; frequency range: 0.1–100,000 Hz). Cyclic voltammetry (CV) was conducted in 0.1 M KCl with [Fe (CN)_6_]^3−^ (5 mM). To verify the successful preparation of the material, collaborative characterization was performed using a UV/vis spectrophotometer (Prism Technology Co., Shanghai, China) and an energy-dispersive X-ray spectrometer (EDS) (JSM 7800F, Tokyo, Japan). The surface morphology of the electrode was visually examined using a scanning electron microscope (SEM) (Zeiss Sigma 300, Obercohen, Germany).

### 2.3. Synthesis of Au@Fc-ConA

The synthesis of the Au@Fc-ConA signal nanoprobe involves three main stages. Initially, Au NPs were synthesized using the classical citric acid reduction method [18]. Then, 100 mL of HAuCl₄·xH₂O (1 mM) was heated to reflux with continuous stirring. Meanwhile, 6.7 mL of trisodium citrate (1 wt%) was added. The mixture was heated and stirred for about 30 min until a stable wine-red color developed, indicating the formation of Au NPs. The solution was then stored at 4 °C for subsequent functionalization.

The second step was the synthesis of Au NPs@ConA [43]. First, 10 μL of TGA was dispersed in 10 mL of Tris-HCl buffer (0.1 M, pH 8.0). To activate the carboxyl groups, 59.72 mg of DMTMM was added, and the mixture was stirred at room temperature for 15 min. Subsequently, 25 µL of ConA (2 mg/mL) was added, and the mixture was stirred continuously for 7 h at pH 7.0 to complete the reaction. The resulting solution was purified by ultrafiltration (molecular weight cutoff: 3000 Da) using 0.01 M phosphate-buffered solution (PBS, pH 7.4), then adjusted to a final volume of 5 mL with 0.1 M Tris-HCl buffer (pH 8.0). Finally, the thiolated ConA was combined with Au nanoparticles (Au NPs) at a 1:2 volume ratio and stirred for 1 h. The final product was stored at 4 °C.

The third step involved the modification of the Fc [44]: 4 mL of Au NPs@ConA solution was centrifuged (8000 rpm, 10 min), and the resulting bottom suspension was resuspended in 0.5 mL of 10% ethanol/water solution (*v*/*v*). Next, 200 μL of n-hexane and 3 μL of Fc was added, and the mixture was incubated in a metal bath for 12 h (25 °C, 600 rpm). Fc molecules were immobilized onto the gold nanoparticles at the hexane/aqueous phase boundary, with the Fc-modified Au NPs remaining in the aqueous phase. The hexane phase was then removed, and the aqueous phase was centrifuged (8000 rpm, 15 min). The pellet was washed three times each with n-hexane and 0.01 M PBS (pH 7.4). Finally, the Au@Fc-ConA nanocomposites were resuspended in PBS to a final volume of 250 μL.

### 2.4. Synthesis of BIP

BIP-modified electrodes (BIP/GCE) were prepared as previously reported [45]: *E. coli* O157:H7 suspension was prepared in PBS solution (0.1 M, pH 6.5) [46] and the functional monomer TE (8 mM) were electropolymerized on a GCE using the CV method (potential range: −0.6–1.0 V vs. SCE; scan rate: 100 mV s^−1^). The modified electrodes were named PTE + *E. coli* O157:H7/GCE. Next, the template was removed by elution with 1 mM CTAB/HAc in a metal bath (37 °C, 400 rpm, 10 min), resulting in the BIP/GCE recognition element for target bacteria detection. At the same time, the non-imprinted polymer (NIP) was built following the equal procedure apart from template bacteria for verification.

### 2.5. Capture and Detection of E. coli O157:H7

The BIP/GCE electrode was initially incubated in a shaking metal bath (37 °C, 250 rpm, 10 min) in 250 μL of 0.01 M PBS solution (pH 7.4) containing a predefined concentration of *E. coli* O157:H7, then rinsed with deionized water to obtain the *E. coli* O157:H7/BIP/GCE. Subsequently, 5.0 μL of Au@Fc-ConA was applied to the electrode surface and incubated at room temperature for 1 h. Unbound nanosignal probes were removed by rinsing with deionized water, resulting in the formation of the final sandwich-type electrode, Au@Fc-ConA/*E. coli* O157:H7/BIP/GCE. The prepared electrode was characterized using differential pulse voltammetry (DPV) [47], performed in 0.01 M PBS at pH 7.4. The DPV experimental potential was set 0.4 to 0.67 V (amplitude value: 0.05 V, pulse width: 0.1 s, width sampling: 0.0167 s). The bacterial concentration was determined by measuring the peak current change (Δ*I*), calculated as Δ*I* = *I* − *I*_0_, where *I* and *I*_0_ represent the peak currents in the presence and absence of target bacteria, respectively.

### 2.6. Selectivity

To assess selectivity, the sensor responses to the target bacteria (*E. coli* O157:H7) and the potential interfering bacteria (*S. aureus*, *S. Paratyphi B*, *L. monocytogenes*, and *E. coli* O6) were compared. The concentration of interfering bacteria was 10 times that of *E. coli* O157:H7 (10^5^ CFU mL^−1^), except for *E. coli* O6, which was present at a concentration 100 times higher.

### 2.7. Real Sample

Milk was used as a real sample to evaluate the sensor’s applicability through a spiked recovery method. Skimmed milk (Yili, China) was purchased from a nearby supermarket and diluted 1:10 with PBS. Subsequently, various concentrations of *E. coli* O157:H7 suspensions were added to the milk samples. Aliquots (250 μL) from each concentration were then subjected to sensor capture and electrochemical detection.

## 3. Results

### 3.1. Characterization of Au@Fc-ConA

**Figure 1:**
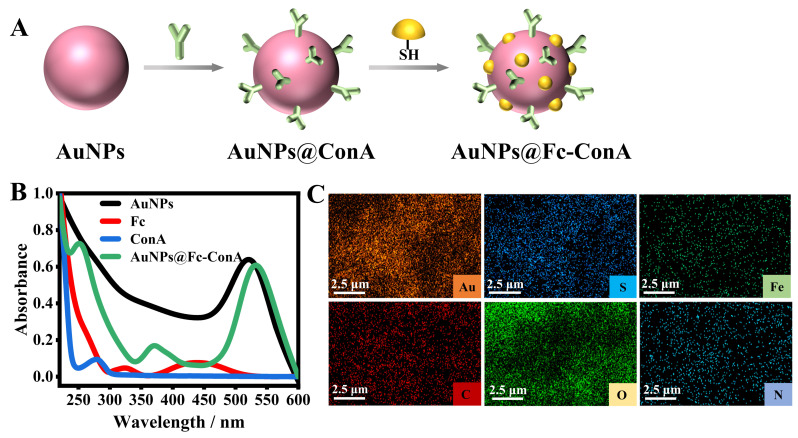
(**A**) Synthesis of the Au@Fc-ConA. (**B**) UV−vis spectra of AuNPs, Fc, ConA, and Au@Fc-ConA. (**C**) EDS elemental mapping of Au@Fc-ConA.

Figure 1A illustrates the synthetic strategy employed to prapare Au@Fc-ConA nanosignal probe, which consist of Au NPs, ConA, and Fc. The thiolated ConA and Fc labels were sequentially attached to the surface of the Au NPs through standard Au-S interactions.

Initially, the interaction between ConA and Fc and Au nanoparticles (Au NPs) was analyzed using ultraviolet–visible (UV–vis) spectroscopy. UV spectra of Au NPs, ConA, Fc, and Au@Fc-ConA were recorded separately to identify their characteristic absorption peaks (Figure 1B). The UV spectrum of Au@Fc-ConA (green line) exhibited a characteristic absorption peak at 530 nm, similar to that of Au NPs alone (blank line), but with a slight red shift. This shift is attributed to changes in surface plasmon resonance (SPR) induced by the coupling of ConA and Fc [18]. Additionally, a blue-shifted absorption peak appeared at 252 nm compared to free ConA (blue line), likely due to conformational changes in the protein upon interaction with Au NPs and Fc, which affected the local environment of its amino acid residues and altered its electron transition properties. Free Fc (red line) displayed two absorption peaks at 330 nm and 445 nm [48,49]. However, after coupling to ConA and Au NPs, the peak positions shifted, and a distinct absorption peak emerged at 370 nm, primarily due to Fc charge transfer [50].

In addition, energy dispersive x-ray spectrometer (EDS) elemental mapping (Figure 1C) revealed that Au@Fc-ConA comprises elements Au, S, Fe, C, O, and N, corresponding to the characteristic elements of Au NPs, Fc, and ConA. These findings confirm the successful fabrication of the Au@Fc-ConA nanoprobe.

### 3.2. Fabrication and Principle of the Sensor

**Figure 2:**
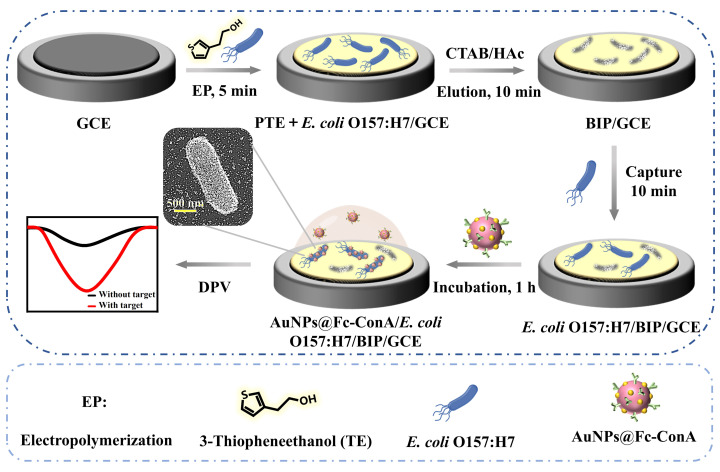
Schematic illustration for the fabrication process and operational principle of the electrochemical sensor for *E. coli* O157:H7 detection.

Figure 2 illustrates the complete manufacturing process for the sensor. The sensing platform consists of two main components: BIP, which serves as a capture probe to selectively identify *E. coli* O157:H7, and Au@Fc-ConA, acting as a signal probe for detection. The TE monomer and template bacteria (*E. coli* O157:H7) are electro-copolymerized onto the surface of a GCE. Subsequently, the template bacteria are removed by elution with a CTAB/HAc solution (1 mM CTAB in 36% acetic acid) at 37 °C for 10 min under constant shaking at 400 rpm. This rapid and straightforward process is completed within 15 min. The resulting BIP exhibits high selectivity, recognizing *E. coli* O157:H7 within 10 min through surface-imprinted sites that complement the target bacteria in shape, size, and chemical functionality [51].

Upon selective capture by the BIP, ConA within the Au@Fc-ConA nanoprobe specifically anchors *E. coli* O157:H7 to the electrode surface. Fc tags on the nanoprobe generate oxidation currents at 0.52 V, proportional to their quantity on the bacterial surface. As the concentration of *E. coli* O157:H7 increases, the current signal rises accordingly, demonstrating a signal-on detection approach for highly sensitive pathogen detection. This method enables reliable detection of low bacterial concentrations.

To further investigate the binding process of the Au@Fc-ConA signaling nanoprobe, scanning electron microscopy (SEM) was employed. After incubation with the nanoprobe, numerous nanoparticles were observed adhering to the surface of captured *E. coli* O157:H7 bacteria. These morphological observations provide direct visual confirmation that the Au@Fc-ConA probes successfully interact with and bind to the bacterial surface following their capture by the BIP on the electrode. The accumulation of nanoparticles on the bacteria’s surface validates the sensor’s specific recognition of *E. coli* O157:H7. This visual evidence further corroborates the sensor’s capability to selectively detect *E. coli* O157:H7 with exceptional specificity, confirming its effectiveness in pathogen detection applications.

### 3.3. Feasibility Assessment and Characterization of the Sensor

**Figure 3:**
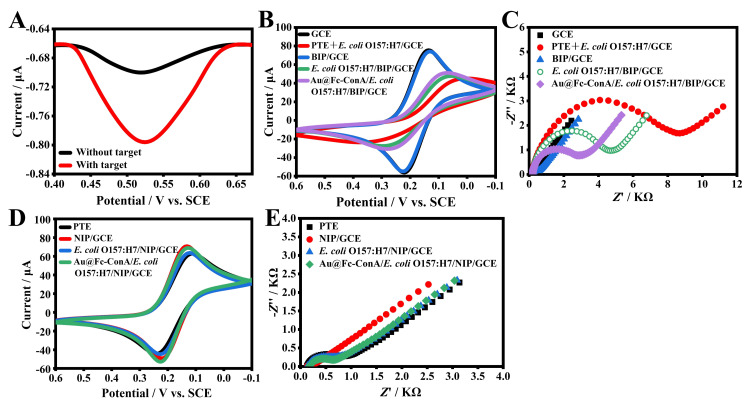
(**A**) DPV signals of the sensor with and without target bacteria in PBS (0.01 M, pH 7.4). (**B**) CV, (**C**) EIS profiles of the GCE during stepwise modification in 0.1 M KCl with [Fe (CN)_6_]^3−/4−^. (**D**) CV and (**E**) EIS profiles of non-imprinted electrodes.

The feasibility of the sensor was evaluated using DPV (Figure 3A). Upon specifically capturing *E. coli* O157:H7, the sensor exhibited a distinct Fc oxidation signal at 0.52 V. In the absence of the target bacteria, a smaller oxidation peak was observed, likely due to the non-specific adsorption of Au@Fc-ConA on the BIP. These findings confirm that the dual-recognition-based sandwich electrochemical sensor is suitable for monitoring *E. coli* O157:H7.

The sensor fabrication was characterized using CV and EIS (Figure 3B,C). The bare electrode exhibited the maximal peak currents (Figure 3B, black line) and the lowest charge-transfer resistance (*R*_ct_) (Figure 3C, solid square). Following the electro-copolymerization of TE with *E. coli* O157:H7, the PTE + *E. coli* O157:H7/GCE exhibited reduced peak currents (Figure 3B, red line) and increased *R*_ct_ (Figure 3C, solid circle), indicating hindered electron transfer due to entrapped bacterial cells. A 10 min CTAB/HAc elution restored the electrode’s characteristics to those of the bare GCE (Figure 3B, blue line; Figure 3C, solid triangle), confirming successful removal of the bacterial template. The BIP/GCE specifically captured target bacteria, as evidenced by the bacterial re-binding, leading to a significant decrease in peak currents (Figure 3B, green line) and increase in charge-transfer resistance (*R*_ct_) (Figure 3C, hollow circle). Further incubation with the Au@Fc-ConA nanoprobe enhanced peak currents (Figure 3B, purple line) and decreased *R*_ct_ (Figure 3C, solid rhombus), attributed to the high conductivity of Au NPs and the electron-bridging role of ConA and Fc.

The control group was also characterized using CV and EIS. In the non-imprinted (NIP) group, *E. coli* O157:H7 was replaced with 0.1 M, pH 6.5 PBS during electropolymerization, while all other steps remained unchanged. As shown in Figure 3D,E, electrode modification resulted in minimal changes in CV and EIS, with negligible variation compared to the experimental group. These results demonstrate the sensor’s applicability in detecting *E. coli* O157:H7.

Figure S1 presents SEM images illustrating the sensor’s fabrication successfully and its interaction with *E. coli* O157:H7. After elution of the polymerized electrode, the surface appears smooth and uniform (Figure S1A), suggesting effective removal of the bacterial template. Upon exposure to *E. coli* O157:H7, bacterial cells are observed on the BIP surface (Figure S1B), indicating the BIP’s capability to capture bacteria. Subsequent incubation with Au@Fc-ConA leads to the presence of dense nanoparticles on the bacterial surface (Figure S1C), attributable to ConA’s binding properties of *E. coli* O157:H7. This characterization provides a direct validation of the feasibility of the sensor.

### 3.4. Quantitative Detection of E. coli O157:H7

**Figure 4:**
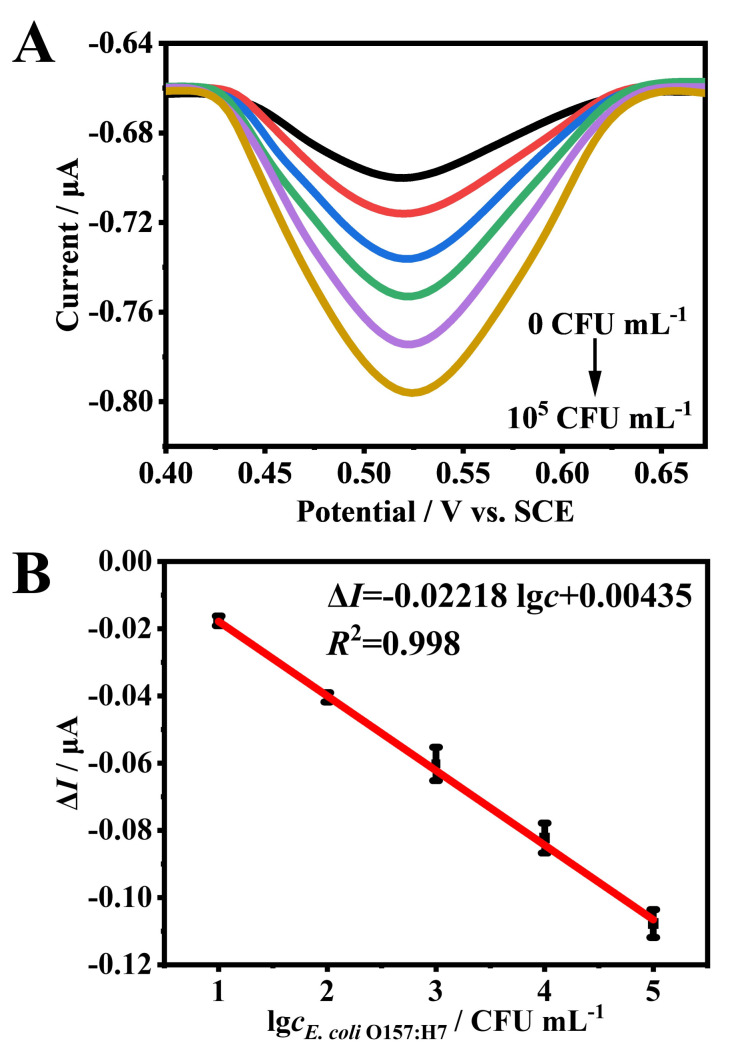
(**A**) DPV of *E. coli* O157:H7 in PBS (0–10^5^ CFU mL^−1^). (**B**) Calibration curve of Δ*I* against logarithmic concentration of *E. coli* O157:H7.

DPV measurements were performed in PBS to quantitatively assess *E. coli* O157:H7. To optimize the peak current, the volume ratio of ConA to Au NPs and the amount of Fc were optimized. As shown in Figure S2, Δ*I* initially increased and decreased with the rising ConA-to-Au NPs ratio, with optimal sensor performance achieved at a ratio of 1:2. Similarly, changes in Fc concentration influenced Δ*I*, with the peak current observed at an optimal Fc dosage of 3 μL. Under these conditions, the peak current increased with higher target bacteria concentrations, demonstrating a signal-on detection mechanism (Figure 4A). Δ*I* exhibited a robust linear correlation with the *E. coli* O157:H7 concentration (10–10^5^ CFU mL^−1^) (Figure 4B), with a regression equation of Δ*I* (μA) = −0.02218 lg*c_E. coli_*_O157:H7_ + 0.00435 (*R*^2^ = 0.998). Using the 3σ/S rule, the sensor’s detection limit was found to be 10 CFU mL^−1^. In comparison to reported electrochemical sensor for *E. coli* O157:H7 (Table S1), the sensitivity of this sensor in terms of detection limit is comparable [9,52,53,54,55,56,57].

### 3.5. Selectivity, Reproducibility, and Stability of the Sensor

**Figure 5:**
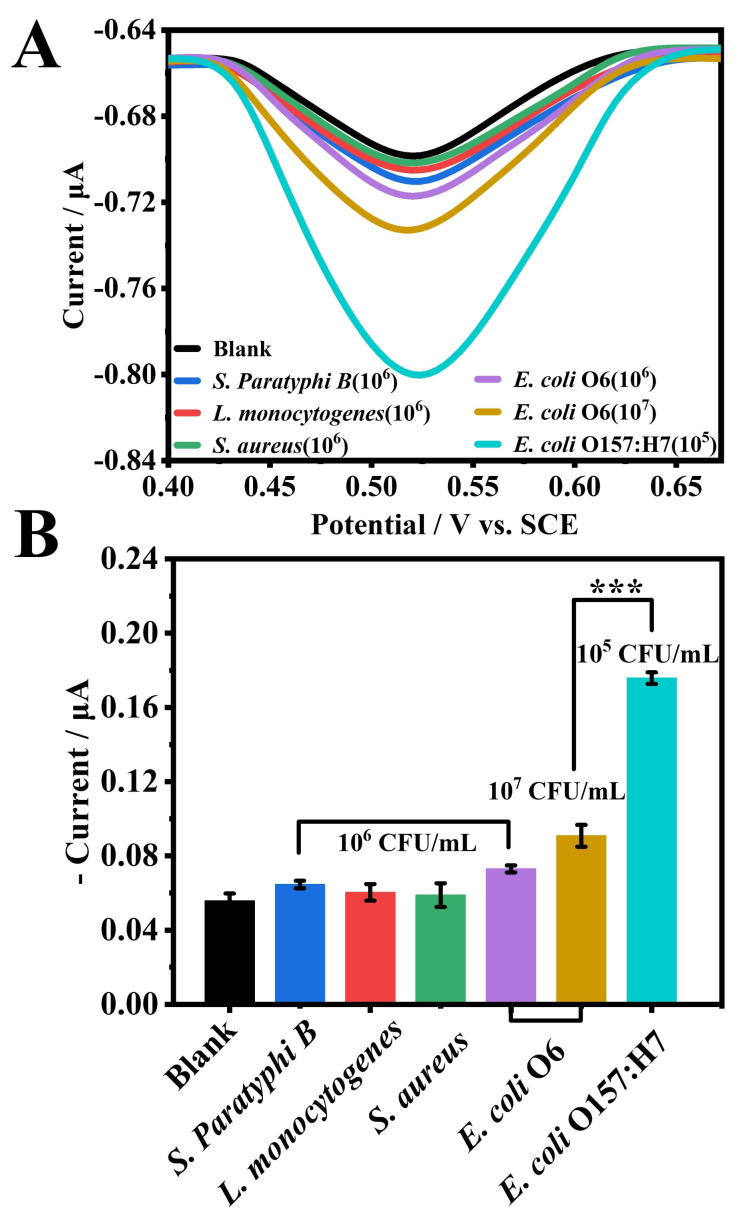
(**A**) DPV current response of the sensor toward *E. coli* O157:H7 and various control bacteria. (**B**) Bar graph showing the statistical analysis of sensor selectivity.

To evaluate the sensor’s selectivity, its response to *E. coli* O157:H7 was compared with that of other common pathogenic bacteria, including *E. coli* O6, *S. Paratyphi B*, *S. aureus* and *L. monocytogenes*, using PBS as a negative control (Figure 5). The current signal for target bacteria was approximately 2.4 to 3 times higher than that for non-target bacteria, whose signals were comparable to the blank control. Notably, even when *E. coli* O6 was present at a concentration 100 times higher than that of the target, the sensor exhibited a 1.9-fold stronger response to *E. coli* O157:H7. The differences in response currents were statistically significant (*p* < 0.001, denoted by ***), underscoring the exceptional selectivity of the proposed sensor. This high selectivity is attributed to the dual recognition involving BIP and ConA, which enables precise distinction between the target bacteria and other bacterial strains. The strong selective binding of *E. coli* O157:H7 by BIP, coupled with the targeted recognition by ConA, ensures effective discrimination of the target pathogen by the sensor.

Reproducibility was assessed by measuring the current response of 10 modified electrodes in parallel, yielding a relative standard deviation (RSD) of 2% (Figure S3A). Additionally, the sensor retained approximately 75% of its initial signal after 15 days at 4 °C (Figure S3B). These findings demonstrate the sensor’s great reproducibility and stability, which are essential for ensuring its reliability and longevity.

### 3.6. Detection of E. coli O157:H7 in Real Samples

Spiked recovery experiments were conducted to evaluate the sensor’s efficacy in detecting *E. coli* O157:H7 in food matrices. Milk was selected as a representative sample and diluted tenfold with sterile PBS to minimize matrix interference. Known concentrations of *E. coli* O157:H7 (10–10^3^ CFU mL^−1^) were introduced to assess the sensor’s detection capability. The average recoveries of *E. coli* O157:H7 ranged from 94.16% to 110.6% (Table 1), indicating that the sensor is a reliable tool for analyzing target bacteria in diluted milk samples.

## 4. Conclusions

In this study, a sandwich-type bacterial electrochemical sensor was developed by integrating a bacteria-imprinted polymer (BIP) for specific recognition with an Au@Fc-ConA nanoprobe for signal amplification. The sensor exhibited remarkable selectivity, effectively distinguishing different *E. coli* serotypes even when the target pathogen was present at a 100-fold lower concentration than interfering species. Additionally, through a simple 10-fold dilution, bacterial concentrations as low as 10 CFU mL^−1^ could be detected in complex milk matrices.

Despite its good performance, the current sensor is limited to the detection of a single pathogen, which may hinder its applicability in complex environments where multiple pathogens coexist. Future research should focus on developing multiplex detection capabilities by designing diverse BIPs and tailored signaling nanoprobes. These advancements will enhance the sensor’s practicality and broaden its application in real-world food safety monitoring.

## Figures and Tables

**Table 1:** Test of *E. coli* O157:H7 in milk (*n* = 3).

Sample	Added (CFU mL^−1^)	Found (mean ± SD/CFU mL^−1^)	Recovery (%)
milk	10	10.17 ± 2.03	101.75
	10^2^	94.16 ± 18.88	94.16
	10^3^	1106.07 ± 305.93	110.6

## Data Availability

The original contributions presented in this study are included in the article. Further inquiries can be directed at the corresponding author.

## Supplementary Materials

The following supporting information can be downloaded at: https://www.mdpi.com/article/10.3390/foods14071099/s1. Figure S1: Scanning electron microscope (SEM) images of differently modified glassy carbon electrodes (GCEs): (A) BIP, (B) enlarged view of *E. coli* O157:H7/BIP; (C) Au@Fc-conA/*E. coli* O157/BIP. Figure S2: The effects of (A) the volume ratio of ConA to Au NPs and (B) the amount of Fc on the current response of the sensor; Figure S3: Reproducibility and storage stability of the sensor; Table S1: Comparison with other sandwich-type electrochemical methods for detecting whole bacterial cells.
