# Supplementary material for: A Dual-Recognition Electrochemical Sensor Using Bacteria-Imprinted Polymer and Concanavalin A for Sensitive and Selective Detection of Escherichia coli O157:H7
Source: Foods. 2025 Mar 21;14(7):1099. doi: 10.3390/foods14071099 (PMC11989128; doi:10.3390/foods14071099)
Supplement: Supplementary file 1 [file foods-14-01099-s001.zip › foods-3515853-supplementary.pdf]

A dual-recognition electrochemical sensor using bacteria-imprinted polymer and concanavalin A for sensitive and selective detection of *Escherichia coli* O157:H7

Xuejie Niu [1], Yuanbing Ma [1], Hui Li [1], Shuang Sun [1], Luoyuan Shi [1], Juan Yan [1], Donglei Luan [1], Yong Zhao [1], and Xiaojun Bian [1,2,3,*]

[1] College of Food Science and Technology, International Research Center for Food and Health, Shanghai Ocean University, Shanghai 201306, China

[2] Laboratory of Quality and Safety Risk Assessment for Aquatic Product on Storage and Preservation (Shanghai), Ministry of Agriculture and Rural Affairs, Shanghai 201306, China

[3] State Key Laboratory of Transducer Technology, Shanghai Institute of Microsystem and Information Technology, Chinese Academy of Sciences, Shanghai, 200050, China

[*] Corresponding authors: Email address: xjbian@shou.edu.cn (X.B.).

Bacterial cultivation

Escherichia coli O157:H7 (*E. coli* O157:H7) and *Escherichia coli* O6 (*E. coli* O6) were inoculated in 10 mL of Luria-Bertani (LB) broth, while *Listeria monocytogenes* (*L. monocytogenes*) was cultivated in Brain Heart Infusion (BHI) broth. *Staphylococcus aureus* (*S. aureus*) and *Salmonella Paratyphi B* (*S. Paratyphi B*) were cultured in Tryptic Soy Broth (TSB). All bacterial strains were incubated at 37 °C with shaking at 200 rpm. All bacterial cells were harvested during exponential growth phase and fixed with 1% formaldehyde. The concentration of each bacterium was determined using the plate count method. Following centrifugation at 10000 rpm for 3 minutes, the supernatant was discarded, and the pellet was re-suspended into fresh phosphate buffer solution by shaking for 1 minute. Ultimately, the harvested cells were prepared as a bacterial suspension of the desired concentration.

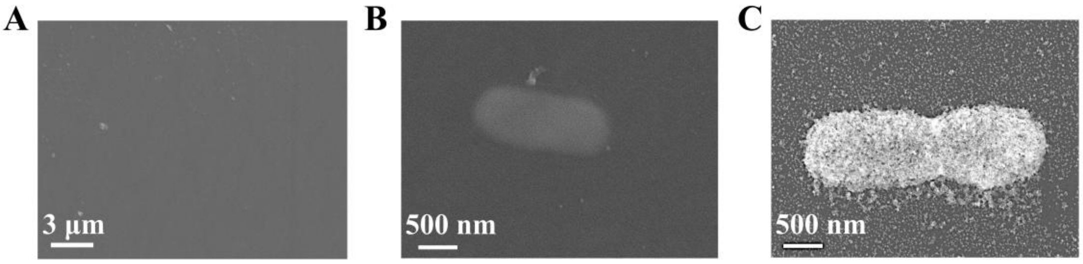

Figure. S1. Scanning electron microscope (SEM) images of differently modified glassy carbon electrodes (GCEs): (A) BIP, (B) enlarged view of E. coli O157:H7/BIP; (C) Au@Fc-conA/E. coli O157/BIP.

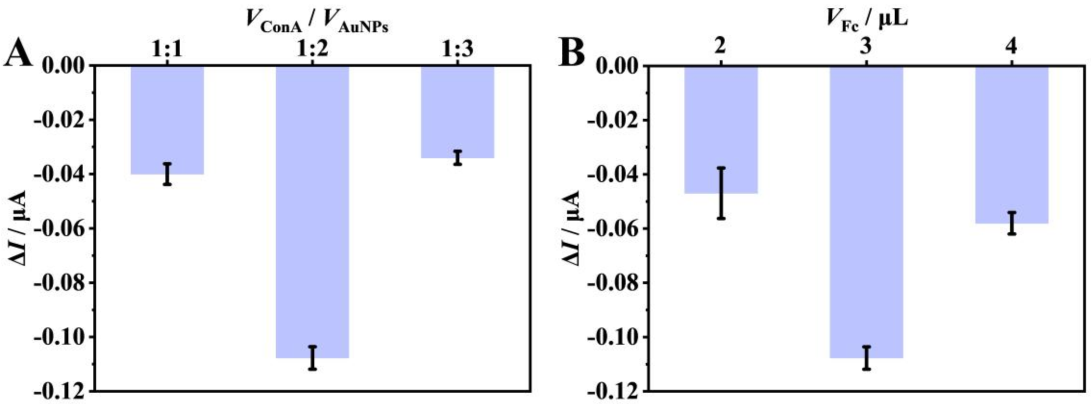

Figure. S2. The effects of (A) the volume ratio of ConA to Au NPs and (B) the amount of Fc on the current response of the sensor.

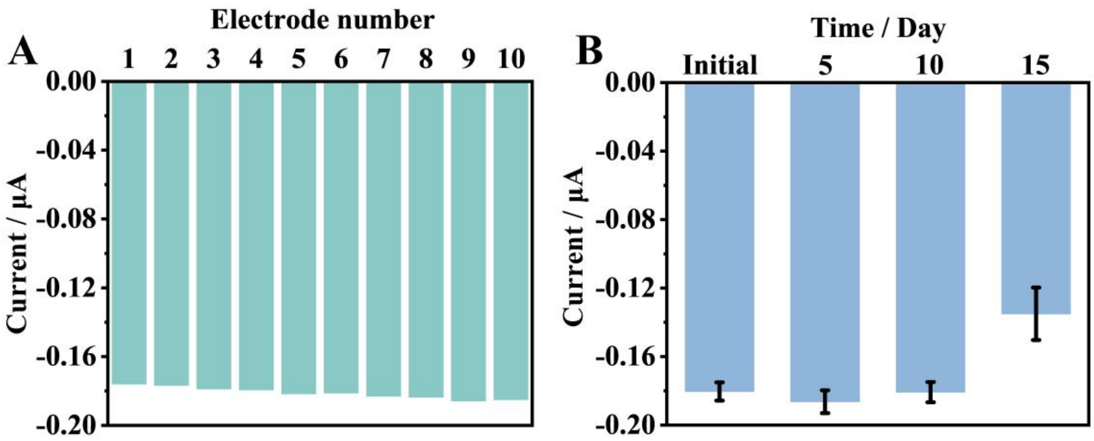

Figure. S3. Reproducibility and storage stability of the sensor.

Table S1. Comparison with other electrochemical methods for detecting *E. coli* O157:H7.

**Target**	**Recognition element**	**Electrode**	**Method**	**Analytical range (CFU/mL)**	**LOD (CFU/mL)**	**Ref.**
E. coli O157:H7	aptamer, antimicrobial peptide	Apt/Au	DPV	10^2-10^7	32	[1]
E. coli O157:H7	mAb	CANF/PB SPE	CA	1.5×10^2-10^6	30	[2]
E. coli O157:H7	antibody	Fe_3O_4@Au MNPs/SPCE	CA	20-2×10^6	20	[3]
E. coli O157:H7	aptamer, 4-MPBA	aptamer/$Ti_3C_2T_X$/GCE	DPV	10-10^5	3	[4]
E. coli O157:H7	aptamer	Au/GCE	EIS	1.5×10^1-1.5×10^5	4	[5]
E. coli O157:H7	mAb	GCE	EIS	3×10^2-3×10^8	7.4	[6]
E. coli O157:H7	mAb	SPE	DPV	10^1-10^9	4	[7]
E. coli O157:H7	BIP, ConA	BIP/GCE	DPV	10-10^5	10	This work

LOD: limit of detection; **Apt**: aptamer; **CA**: conoamperometry; **mAb**: monoclonal antibody; **CANF**: cellulose acetate nanofiber; **PBSPE**: paper-based screen-printed electrode; **SPCE**: screen-printed carbon electrodes; **4-MPBA**: 4-mercaptophenylboronic acid; **SPE**: screen printed electrode

References

1. Bu, S.; Wang, K.; Li, Z.; Wang, C.; Hao, Z.; Liu, W.; Wan, J. An electrochemical biosensor based on methylene blue-loaded nanocomposites as signal-amplifying tags to detect pathogenic bacteria. Analyst 2020, 145, 4328-4334, doi:10.1039/d0an00470g.
2. Zhang, Z.; Du, M.; Cheng, X.; Dou, X.; Zhou, J.; Wu, J.; Xie, X.; Zhu, M. A disposable paper-based electrochemical biosensor decorated by electrospun cellulose acetate nanofibers for highly sensitive bio-detection. Analyst 2024, 149, 2436-2444, doi:10.1039/d4an00164h.
3. Bazsefidpar, S.; Freitas, M.; Pereira, C.R.; Gutiérrez, G.; Serrano-Pertierra, E.; Nouws, H.P.A.; Matos, M.; Delerue-Matos, C.; Blanco-López, M.C. Fe3O4@Au Core–Shell Magnetic Nanoparticles for the Rapid Analysis of E. coli O157:H7 in an Electrochemical Immunoassay. Biosensors 2023, 13, doi:10.3390/bios13050567.
4. Dai, G.; Li, Y.; Li, Z.; Zhang, J.; Geng, X.; Zhang, F.; Wang, Q.; He, P. Zirconium-Based Metal–Organic Framework and Ti3C2Tx Nanosheet-Based Faraday Cage-Type Electrochemical Aptasensor for Escherichia coli Detection. ACS Applied Nano Materials 2022, 5, 9201-9208, doi:10.1021/acsanm.2c01548.
5. Li, Z.; Zhang, X.; Qi, H.; Huang, X.; Shi, J.; Zou, X. A novel renewable electrochemical biosensor based on mussel-inspired adhesive protein for the detection of Escherichia coli O157:H7 in food. Sensors and Actuators B: Chemical 2022, 372, doi:10.1016/j.snb.2022.132601.
6. Duan, X.; Shi, X.; He, Z.; Chen, H.; Shi, Z.; Zhao, Z.; Chen, H.; Yu, M.; Guo, C. Conducting polymer functionalized Cu-metal organic framework–based electrochemical immunosensor for rapid and sensitive quantitation of Escherichia coli O157:H7. Microchimica Acta 2024, 191, doi:10.1007/s00604-024-06807-1.
7. Ramesh, M.; Umamatheswari, S.; Vivek, P.M.; Sankar, C.; Jayavel, R. Synthesis of silver-bismuth oxide encapsulated hydrazone functionalized chitosan (AgBi2O3/FCS) nanocomposite for electrochemical sensing of glucose, H2O2 and Escherichia coli O157:H7. International Journal of Biological Macromolecules 2024, 264, doi:10.1016/j.ijbiomac.2024.130533.
